# The First Extracellular Domain Plays an Important Role in Unitary Channel Conductance of Cx50 Gap Junction Channels

**DOI:** 10.1371/journal.pone.0143876

**Published:** 2015-12-01

**Authors:** Xiaoling Tong, Hiroshi Aoyama, Swathy Sudhakar, Honghong Chen, Brian H. Shilton, Donglin Bai

**Affiliations:** 1 Department of Physiology and Pharmacology, University of Western Ontario, London, Ontario, Canada; 2 Graduate School of Pharmaceutical Sciences, Osaka University, Osaka, Japan; 3 Department of Biochemistry, University of Western Ontario, London, Ontario, Canada; Universidade Federal do ABC, BRAZIL

## Abstract

Gap junction (GJ) channels provide direct passage for ions and small molecules to be exchanged between neighbouring cells and are crucial for many physiological processes. GJ channels can be gated by transjunctional voltage (known as V_j_-gating) and display a wide range of unitary channel conductance (γ_j_), yet the domains responsible for V_j_-gating and γ_j_ are not fully clear. The first extracellular domain (E1) of several connexins has been shown to line part of their GJ channel pore and play important roles in V_j_-gating properties and/or ion permeation selectivity. To test roles of the E1 of Cx50 GJ channels, we generated a chimera, Cx50Cx36E1, where the E1 domain of Cx50 was replaced with that of Cx36, a connexin showing quite distinct V_j_-gating and γ_j_ from those of Cx50. Detailed characterizations of the chimera and three point mutants in E1 revealed that, although the E1 domain is important in determining γ_j_, the E1 domain of Cx36 is able to effectively function within the context of the Cx50 channel with minor changes in V_j_-gating properties, indicating that sequence differences between the E1 domains in Cx36 and Cx50 cannot account for their drastic differences in V_j_-gating and γ_j_. Our homology models of the chimera and the E1 mutants revealed that electrostatic properties of the pore-lining residues and their contribution to the electric field in the pore are important factors for the rate of ion permeation of Cx50 and possibly other GJ channels.

## Introduction

Gap junction (GJ) channels are intercellular channels, providing a direct passage for ions and small molecules, up to about 1 kilodalton in size, between adjacent cells. Each gap junction channel is formed by the docking of two hemichannels at their extracellular domains. Hemichannels are homo- or hetero-oligomeric proteins of 20 (in mouse) or 21 (in human) homologous connexins [1,2]. All connexins share similar structural topology with four transmembrane domains (M1–M4) linked by the first and second extracellular loops (E1 and E2, respectively) and one cytoplasmic loop with both amino-terminus (NT) and carboxyl-terminus residing in the cytoplasm. The E1 and E2 domains not only serve as the key docking sites to “glue and seal” two hemichannels at the extracellular medium, but also form part of the GJ channel wall exterior and interior (pore lining). In theory the pore lining residues, including those residues from the extracellular domains, are uniquely positioned to facilitate/limit permeation of ions/molecules and to sense transjunctional voltage (V_j_), which can trigger V_j_-dependent gating (or V_j_-gating), a common property found in all characterized GJ channels [3,4,5].

Experimental evidence supports the idea that part of the first extracellular domain/loop (E1) of several connexins lines a portion of the GJ pore. First, recombinant expression studies with exchanging the entire E1 domain between Cx32 and Cx26 resulted in altered V_j_-gating properties [6,7]. Similarly, switching E1 domains between Cx40 and Cx43 [8], Cx32 and Cx43 [9,10,11], Cx32 and Cx46 [12] or Cx36 and Cx43 [13] were also found to change V_j_-gating properties, unitary channel conductance (γ_j_) or cation/anion preference. Second, point mutations of the residues, especially charged residues, in the E1 of Cx26, Cx32, Cx36, Cx43, Cx46 and Cx50 were found to alter the resultant channel properties [7,13,14,15,16,17,18]. Third, using substituted cysteine accessibility method (SCAM) the initial part of E1 domain was proposed to line the pore of Cx46, Cx50 and possibly other GJ hemichannels [14,16,19]. Finally, high resolution crystal structure analysis of the Cx26 GJ channel showed that E1 domain lines part of the Cx26 GJ pore [20,21]. Sequence alignment of the E1 domains of all known connexins revealed that this domain displays the highest sequence identity among all connexin domains [22], suggesting that the E1 domains of these connexins are likely share similar structures to that of Cx26.

**Fig 1:**
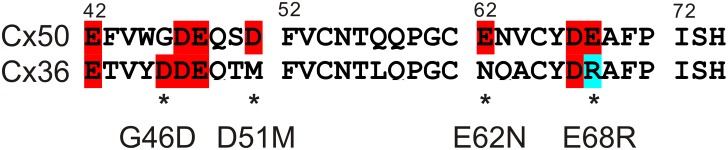
Sequence alignment of the first extracellular domain of Cx50 and Cx36. The first extracellular domains (E1) of mouse Cx50 (42–74) and Cx36 (43–75) are aligned. Ten out of thirty-three (10/33) residues are different between these two connexins, including four residues with charge changes (asterisks), G46D, D51M, E62N and E68R. The naming of these mutants was according to the residue number of Cx50. Negatively charged residues (red) and positively charge residue (blue) are highlighted.

It was well characterized that the lens connexin, Cx50, formed one of the largest GJ channels in terms of the γ_j_ (~200 pS) and displayed prominent V_j_-gating [23,24], while the neuronal connexin, Cx36, formed one of the lowest γ_j_, often beyond detection, and showed very weak V_j_-gating [25,26,27,28]. We hypothesize that the γ_j_ and V_j_-gating properties of these two quite distinct connexin channels are determined in part by the differences in their respective E1 domains. To test this we generated a chimera Cx50Cx36E1, in which the E1 of Cx50 was replaced with the corresponding E1 of Cx36 (Fig 1), and performed dual patch clamp analysis on the macroscopic and unitary channel currents. In addition we also studied four point mutations involving amino acid side chain charge changes in the E1 individually, namely G46D, D51M, E62N and E68R. Our data indicate that Cx50Cx36E1, G46D, E62N, and E68R are capable of forming functional GJ channels with little/modest changes in V_j_-gating properties, indicating that Cx36 E1 domain and several key residues are unlikely to be responsible for its uniquely low γ_j_ and V_j_-gating sensitivity. The D51M mutant failed to form functional GJ channels and displayed an abnormal localization. Unexpectedly, all the point mutant channels showed an elevated γ_j_, indicating the E1 domain in Cx50 acts as a partial ion permeation barrier. Our homology structure models of the chimera and the mutants indicate that several residues of the E1 domain face the pore lumen and the electrostatic properties of these pore-lining residues play an important role in regulating the rate of ion permeation of Cx50 GJ channel.

## Materials and Methods

### Construction of Cx50 mutants

Mouse Cx50 cDNA was inserted into a mammalian expression vector pIRES2-EGFP as described [29]. Mouse Cx36 cDNA was subcloned into pIRES2-EGFP in EcoRI restriction site. The chimera Cx50Cx36E1 was constructed by replacing Cx50 E1 domain (residues 42 ~ 74) with the corresponding domain of Cx36 (residues 43 ~ 75) (see Fig 1). Cx50 vector was used as a template to generate the chimera and individual point mutants G46D, D51M, E62N and E68R with a Quick-Change site directed mutagenesis kit (Stratagene, La Jolla, CA). Primers for the chimera and mutants are listed as follows except G46D, which was described earlier [29]:

Cx50Cx36E1

Forward: 5’CTCGGGACAGCAGCGGAGACGGTGTACGATGATGAGCAGACCATGTTTGT

GTGCAACACCCTACAGCCCGGCTGTAACCAGGCCTGCTATGACCGCGCCTTTCCCATCTCC

CATATCCGCCTCTGGGTGCTGCAG 3’

Reverse: 5’CTGCAGCACCCAGAGGCGGATATGGGAGATGGGAAAGGCGCGGTCATAGCA

GGCCTGGTTACAGCCGGGCTGTAGGGTGTTGCACACAAACATGGTCTGCTCATCATCGTAC

ACCGTCTCCGCTGCTGTCCCGAGG 3’

D51M Forward: 5’ GGGCGATGAGCAATCTATGTTTGTATGCAACACCCAGC 3’

Reverse: 5’ GCTGGGTGTTGCATACAAACATAGATTGCTCATCGCCC 3’

E62N Forward: 5’GCCAGGCTGTAATAATGTCTGCTACGATGAGG3'

Reverse: 5’ CCTCATCGTAGCAGACATTATTACAGCCTGGC 3'

E68R Forward: 5’ GTCTGCTACGATAGGGCCTTTCCCATC 3’

Reverse: 5’ GATGGGAAAGGCCCTATCGTAGCAGAC 3'

### Cell culture and transient transfection

Mouse neuroblastoma (N2A) cells were purchased from American Type Culture Collection (ATCC, Manassas, VA) and cultured with Dulbecco’s modified Eagle’s medium (DMEM) containing 10% fetal bovine serum (FBS) [29]. Before transfection, cells were plated in 35 mm dishes and the confluence was around 50% after overnight culture. 1 μg Cx50 construct, the chimera, or one of the mutant vectors was transfected with 2 μl X-tremeGENE HP DNA Transfection Reagent (Roche Applied Sciences, Indianapolis, IN). Cells were cultured for 24 hours after transfection and replated on to glass coverslips ~1–3 hours prior patch clamping recording.

### Immunolabeling with Cx50 antibody

HeLa cells were cultured in DMEM supplemented with 10% FBS. Mouse Cx50-IRES-GFP or D51M-IRES-GFP was transfected with X-treme GENE HP DNA transfection reagent. After overnight culture the cells were fixed with 4% paraformaldehyde for 10 minutes at room temperature and then the cells were permeabilized with 0.1% Triton X-100 in PBS for 10 minutes and blocked with 3% BSA (Sigma) in PBS for 2 hours at room temperature or overnight at 4°C. Transfected HeLa cells were incubated with goat polyclonal anti-Cx50 C-terminal peptide antibody (Santa Cruz Biotechnology) with 1:400 dilution in blocking solution for 1 hour at room temperature. After washing three times with PBS, the cells were incubated with secondary antibody conjugated with Alex Flour594 (Invitrogen) at a dilution of 1:500 in blocking solution for 30 minutes. The cell nuclei were stained with bisBenimide H33258 (Sigma, 0.01 μg / μl in PBS) for two minutes. Fluorescent images were obtained from a CCD camera (Media Cybernetics, Rockville, MD) mounted on a fluorescent microscope (Olympus BX51) using a 40x water immersion lens. Fluorescent images and/or DIC images were superimposed with ImageMaster software to show the localizations of Cx50 and D51M.

### Electrophysiological recording

The V_j_-gating property of cell pairs expressing either Cx50 or its mutants was measured by dual whole-cell voltage-clamp technique as described earlier [24,29,30]. Briefly, the transfected cells were replated on glass coverslips and then transferred to a recording chamber on an inverted microscope (Leica DM IRB, Wetzlar, Germany) filled with extracellular fluid (ECF) at room temperature. The composition of ECF is (in mM): 140 NaCl, 2 CsCl, 2 CaCl_2_, 1 MgCl_2_, 5 Hepes, 4 KCl, 5 D-glucose, 2 Pyruvate, pH 7.2. Paired GFP-positive cells were patched by two glass micropipettes (pipette resistance 2–4 MΏ) which were filled with intracellular fluid (ICF) containing (in mM): 130 CsCl, 10 EGTA, 0.5 CaCl_2_, 3 MgATP, 2 Na_2_ATP, 10 Hepes, pH 7.2. To test transjunctional voltage-dependent gating in an isolated cell pair, one cell of the pair was clamped at 0 mV while the apposed cell was administrated with a series of voltage pulses (7 seconds in duration) from ± 20 mV to ± 100 mV in a 20 mV increment. The macroscopic junctional currents (I_j_s) were amplified with Axopatch 200B amplifiers with a low-pass filter (cut-off frequency 1 kHz) and digitalized at 10 kHz sampling rate via an ADDA converter (Digidata 1322A, Molecular devices, Sunnyvale, CA).

### Homology structure modeling and electrostatic analysis

The sequence of mouse Cx50 was aligned with that of Cx26 for homology structure models. High sequence identity is observed in these two proteins (overall 49% and on the structurally resolved domains 57%). The Cx26 crystal structure (2ZW3) [20] was used as a template for the Cx50 structure. When a Cx50 residue replacement in the structure caused an abnormal inter-atomic contact, this was adjusted by hand initially in COOT and then revised by CNS energy refinement. After the energy refinement, structural validity of the model was inspected manually as described earlier [31,32]. Adaptive Poisson-Boltzmann Solver (APBS) [33] and PDB2PQR server (http://nbcr-222.ucsd.edu/pdb2pqr_1.8/) were used to calculate the electrostatic potentials of all atoms in the protein and their influence in the pore centre. The APBS parameters were set as described previously [20]. PyMOL program was used for the diameter measurements and the structure presentations [34]. To obtain the electrostatic potentials in the centre of the channel, the centre of the pore in the homology model was first aligned with the x-axis using PoreWalker [35] and then rotated 90° to align with the z-axis. Electrostatic potentials were re-calculated using APBS as implemented in PyMOL v1.7.4.1, and the values for the grid points along the z-axis were extracted from the DX file using OpenDX with “potential.net”, a modified version of the sample program “PlotLine.net” (bundled with OpenDX 4.4.4). “Potential.net” is a text file provided in the Supporting Information (S1 File) and can be used once it is edited to provide the path to the DX file.

### Data analysis

To minimize the influence of series resistance on V_j_-gating properties, only those cell pairs with ≤ 5 nS junctional conductance (G_j_) were selected for Boltzmann fitting analysis [36]. For each current trace, the normalized steady-state conductance (G_j,ss_) was obtained by normalizing the steady state current to the peak current. The dependence of G_j,ss_ to positive or negative V_j_ was plotted and fitted with a two-state Boltzmann equation independently:

$${\text{G}}_{\text{j},\text{ss}}\;=\frac{{\text{G}}_{\text{max}}-{\text{G}}_{\text{min}}\;}{1+{\text{ e}}^{\text{A}\left({\text{V}}_{\text{j}}\;-{\text{ V}}_{\text{o}}\right)}}+{\text{G}}_{\text{min}}$$

V_0_ is the voltage at which the conductance is reduced by half [(G_max_—G_min_)/2], G_max_ is the maximum normalized conductance, G_min_ is the normalized voltage-insensitive residual conductance, and parameter *A*, describing the slope of the fitted curve, which reflects the V_j_ sensitivity of the GJ channels.

To record unitary channel current (i_j_), cell pairs with one or two operational channels were obtained [29]. The amplitude of i_j_s was measured directly using Clampfit9 after digital filtering and plotted to corresponding V_j_s. The i_j_—V_j_ plot was fitted by linear regression through the origin of the coordinates. The slope of the linear regression line is defined as the slope unitary conductance (γ_j_).

## Results

### Cx50Cx36E1 channel was functional and showed a similar G_j,ss_-V_j_ relationship with Cx50

**Fig 2:**
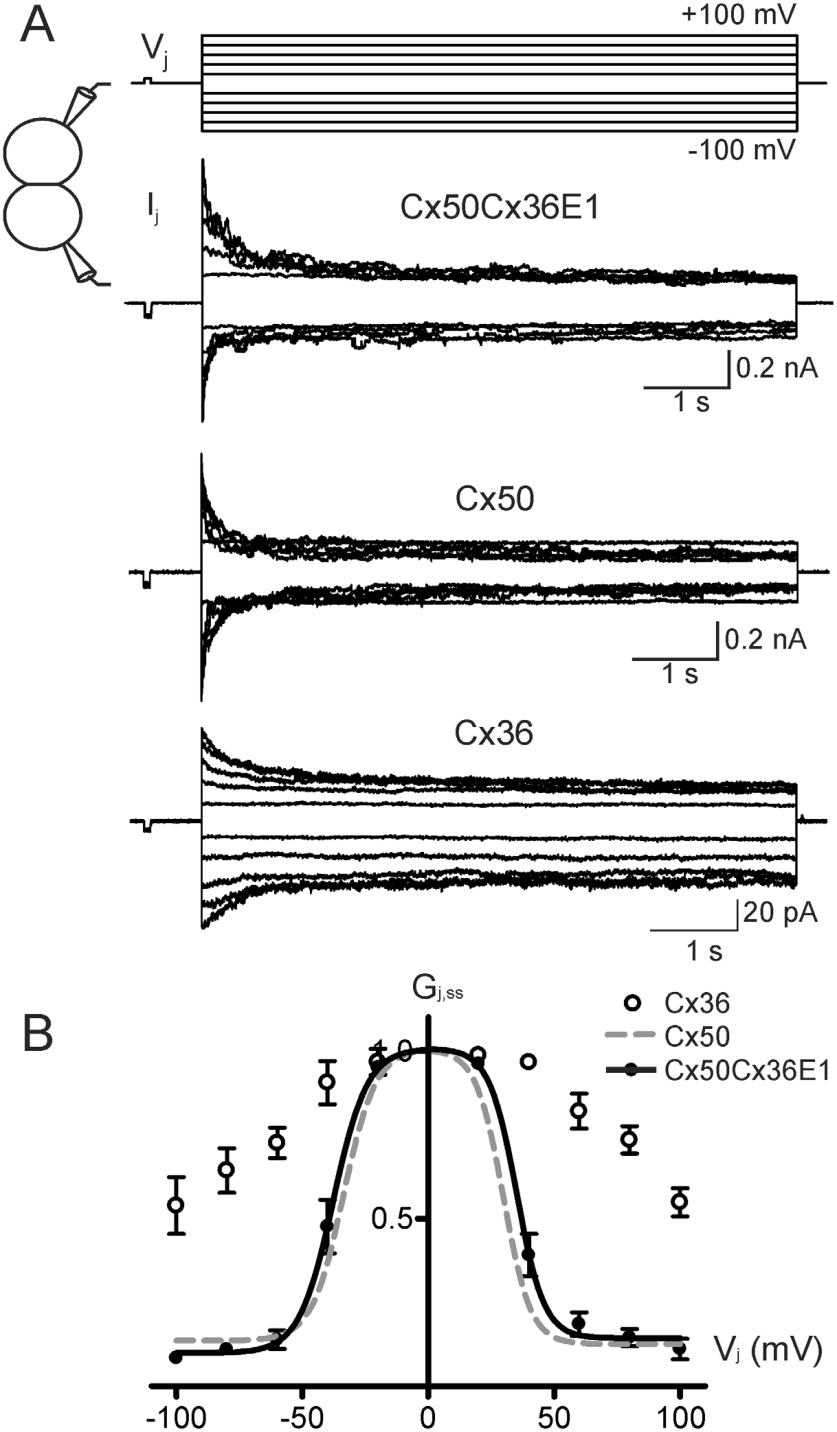
Macroscopic V_j_-gating properties of Cx50Cx36E1 gap junction channels. **A)** V_j_ pulses from ± 20 mV to ± 100 mV in a 20 mV increment were applied to one cell of the cell pair expressing Cx50Cx36E1 and macroscopic transjunctional currents (I_j_s) recorded from the other cell are presented. For comparison, I_j_s from cell pairs expressing Cx50 or Cx36 are also shown. **B)** Normalized G_j,ss_ of Cx50Cx36E1 (filled circles) and Cx36 (open circles) at different V_j_s were plotted. The smooth black lines represent the best fitting curves of the averaged data from Cx50Cx36E1 (n = 4) to a two-state Boltzmann function. Smooth long dashed lines are Boltzmann fits of G_j,ss_−V_j_ plots of the Cx50 channels [29]. We were unable to use Boltzmann equation to fit the data obtained from Cx36 channels.

N2A cell pairs successfully expressing the chimera, Cx50Cx36E1, were chosen for double patch clamp analysis. In response to positive and negative V_j_ pulses (Fig 2A), the macroscopic junctional currents (I_j_s) showed a mirror symmetrical and V_j_-dependent deactivation (Fig 2A). The deactivation of Cx50Cx36E1 I_j_s was absent at V_j_s of ± 20 mV and was gradually increased with the increase in the absolute values of V_j_s. The apparent rates of deactivation were also increased with the values of V_j_s (Fig 2A). These properties were qualitatively similar to those observed in Cx50 channels, but unlike those of Cx36 (Fig 2A). The normalized steady-state junctional conductance (G_j,ss_) to V_j_ plots of Cx50Cx36E1 (filled circles) were fitted nicely to Boltzmann equations (smooth black lines) similar to those of Cx50 channels (smooth long dashed grey lines). These were very different from the plots of Cx36 (Fig 2B, open circles), which could not be fitted by the Boltzmann equation. The fitting parameters in Table 1 indicate that the slope *A* and G_min_ values of Cx50Cx36E1 are not significantly different from those of Cx50, but the V_0_ values of the chimera channel are slightly higher than those of Cx50 (P < 0.001, see Table 1). Given that there are 10 amino acid residues different in the E1 domains between Cx50 and Cx36, including 4 charge changes (Fig 1), we were surprised to observe little change in macroscopic V_j_-gating properties of Cx50Cx36E1 to the wildtype Cx50.

**Table 1:** Boltzmann fitting parameters for Cx50 E1 chimera and mutants.

connexin	V_j_ polarity	G_min_	V_0_	*A*
Cx50^†^	+	0.13 ± 0.01	29.8 ± 0.8	0.18 ± 0.01
Cx50^†^	-	0.14 ± 0.01	33.6 ± 1.0	0.16 ± 0.02
Cx50Cx36E1	+	0.14 ± 0.02	35.1 ± 1.7***	0.18 ± 0.05
Cx50Cx36E1	-	0.10 ± 0.04	37.8 ± 1.4***	0.15 ± 0.04
G46D^†^	+	0.13 ± 0.02	31.1 ± 1.5	0.15 ± 0.02**
G46D^†^	-	0.12 ± 0.02	30.0 ± 1.2***	0.15 ± 0.02
E62N	+	0.20 ± 0.02***	32.4 ± 2.2*	0.14 ± 0.03*
E62N	-	0.18 ± 0.03*	31.5 ± 2.8	0.11 ± 0.03**
E68R	+	0.19 ± 0.03	39.1 ± 2.6**	0.08 ± 0.02**
E68R	-	0.19 ± 0.03	37.4 ± 2.5	0.13 ± 0.04

Data are presented as mean ± SEM and V_0_ are absolute values. Student’s *t*-test was used to compare the Boltzmann fitting parameters of the mutants against those of the wild-type Cx50 with the same V_j_ polarity. The number of asterisks indicate the statistical difference level:

* p < 0.05,

** p < 0.01,

*** p < 0.001.

^†^Data are obtained from [29]. Number of cell pairs used for this analysis is as follows: Cx50, n = 6; Cx50Cx36E1, n = 4; G46D, n = 6; E62N, n = 6; E68R, n = 4.

### The unitary channel conductance (γ_j_) of Cx50Cx36E1 channel was reduced

**Fig 3:**
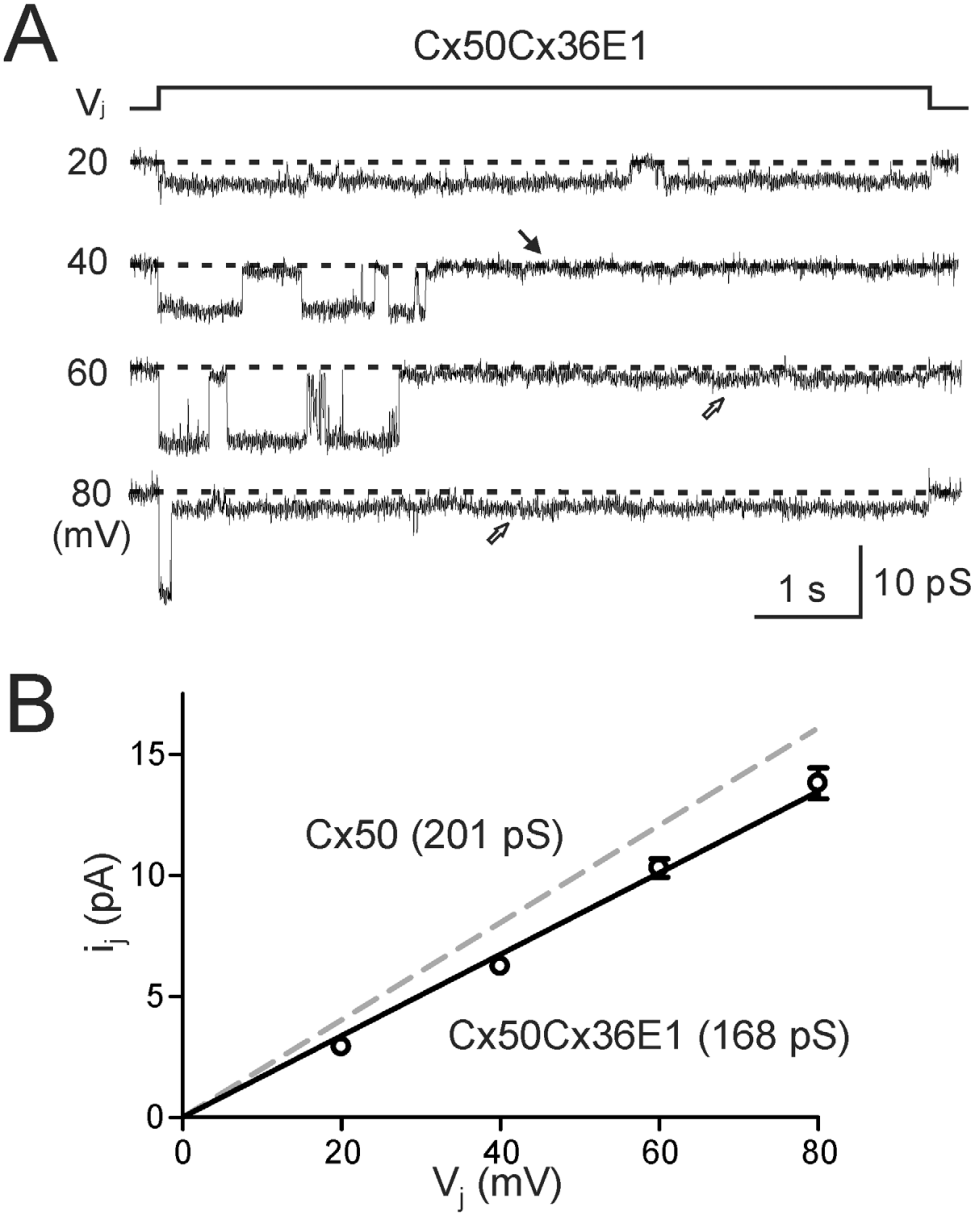
Cx50Cx36E1 unitary channel conductance was lower than that of Cx50. **A)** Representative single channel currents (i_j_s) of Cx50Cx36E1 are illustrated in response to different V_j_s as indicated. Depending on the V_j_ pulse amplitude, the i_j_s of Cx50Cx36E1 channel are dwelled in either the main conducting, a long lived subconductance state (open arrows) or a fully closed state (black arrow). **B)** Average single channel slope conductance (γ_j_) of the main conducting state of Cx50Cx36E1 (168 ± 3 pS, n = 7) was significantly lower than that of Cx50 (201 ± 2 pS, P < 0.01). The slope γ_j_ of Cx50 is from [29].

To evaluate the unitary channel properties of Cx50Cx36E1, we studied cell pairs displaying clear unitary channel currents (i_j_s). As shown in Fig 3A, i_j_s of single Cx50Cx36E1 channel were obtained. At V_j_ of 20 mV, the channel mostly stayed in the open state throughout the V_j_ pulse. However, at higher V_j_s the channel was likely to be in an open state in the initial portion of the V_j_ pulse and then the channel mostly transitioned into a long lived subconductance or closed state. These single channel characteristics are qualitatively similar to those observed in wildtype Cx50 channel, except that the slope γ_j_ of the main open state of Cx50Cx36E1 (168 ± 3 pS, n = 7, Fig 3B) was significantly lower than that of Cx50 (201 ± 2 pS, n = 8, P < 0.001). The average of subconductance state conductance at ± 80 mV V_j_ (γ_j,sub_ = 26 ± 3 pS, n = 4) was also significantly lower than that of Cx50 (39 ± 1 pS, n = 6, P < 0.01), indicating that the chimera channel is less favorable for ion permeation for both main and subconducting states. The functional changes in the chimera could be due to one or more of the different amino acid residues in the E1 domain.

### Functional analysis of the Cx50 point mutants in the E1 domain

**Fig 4:**
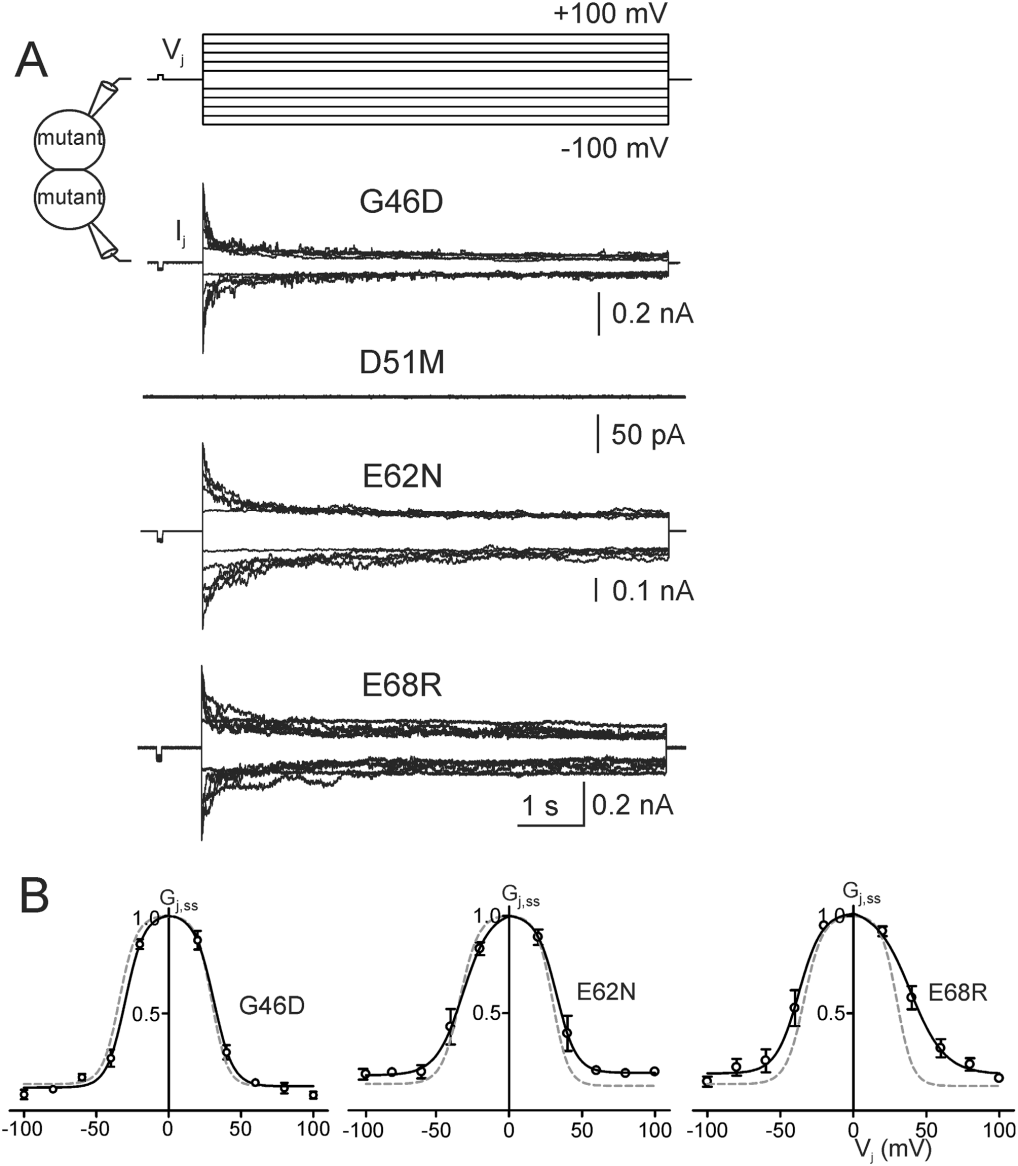
Functional status and V_j_-gating properties of Cx50 E1 mutants. **A)** Representative macroscopic junctional currents (I_j_s) of homotypic G46D, D51M, E62N and E68R channels are shown in response to the V_j_s shown. G46D, E62N and E68R were capable of forming functional homotypic GJ channels and strongly gated by V_j_s similar to those of Cx50. However, D51M failed to form any functional GJ channels. **B)** G_j,ss_-V_j_ relationships of G46D (n = 6), E62N (n = 6) and E68R (n = 4) were constructed and were fitted to Boltzmann functions (smooth black lines). The fitting curves of Cx50 (long dashed grey lines) are shown for comparison. The Boltzmann fitting curves of Cx50 and G46D are from [29].

Sequence alignment of the E1 domain of Cx50 and Cx36 reveals that there are 10 different amino acid residues (Fig 1). Four of them involve charge changes (G46D, D51M, E62N and E68R, see Fig 1). To test the roles of these charge changed mutants, we generated these point mutants individually and tested their ability to form functional channels and their channel properties. All of these mutants except D51M were able to form functional GJ channels (Fig 4A). The G_j,ss_—V_j_ plots of G46D, E62N and E68R could be described by the Boltzmann equations with either similar parameters as those of Cx50 or some moderate changes in slope (*A*), V_0_ and G_min_ for these mutant channels (Fig 4, Table 1).

**Fig 5:**
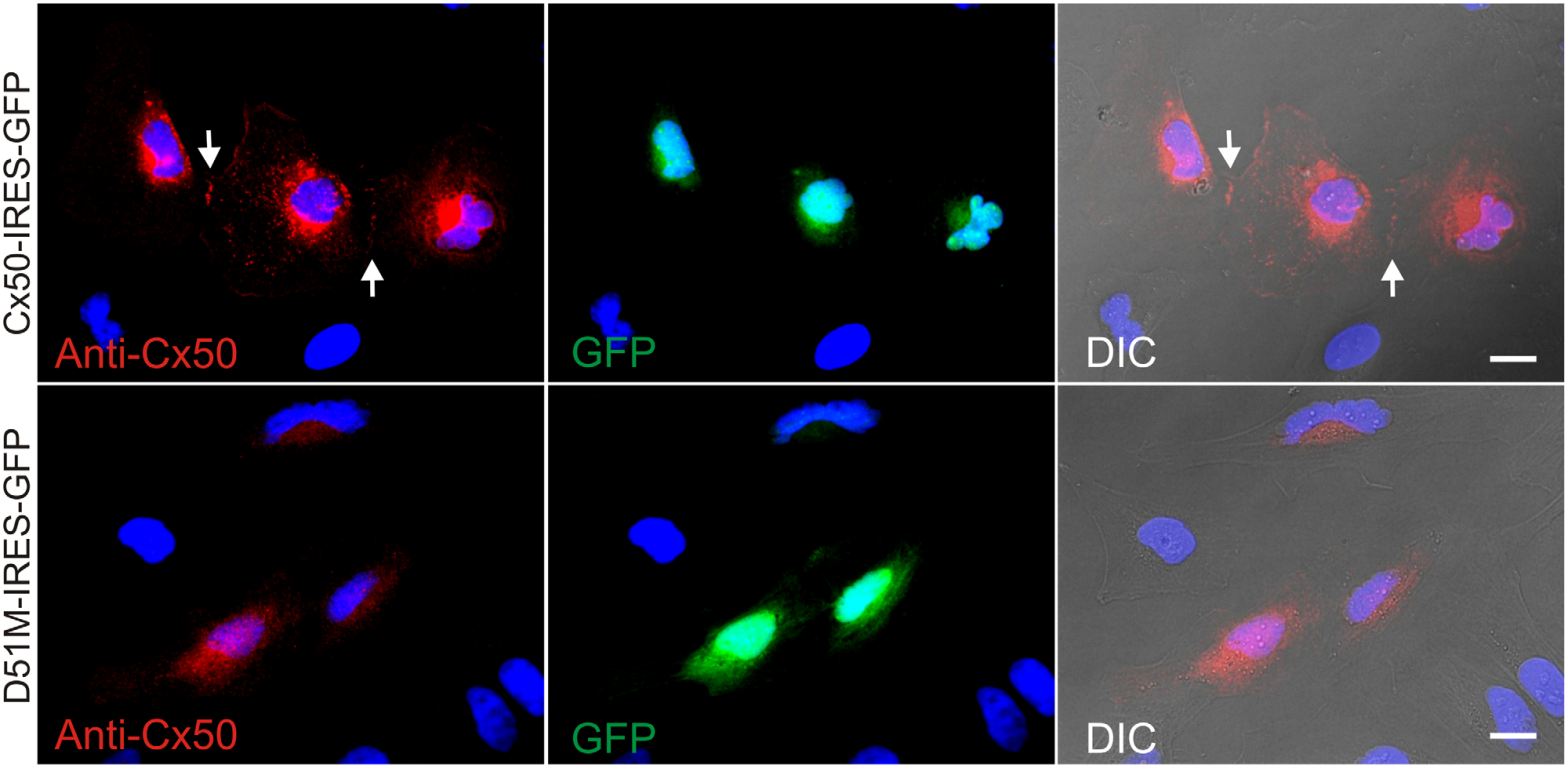
Cx50 D51M diffusely distributed in intracellular compartments without visible gap junction plaque-like structures at cell-cell interfaces. Anti-Cx50 C-terminal peptide antibody labeling (red, left panels) are shown. Cells expressing Cx50-IRES-GFP (top panels) showed punctate localization around the nuclei, intracellular compartments as well as at cell-cell interfaces forming GJ plaque-like structures (arrows), while in the cells expressing D51M-IRES-GFP (bottom panels), the mutant was localized diffusely in the cytosol and no GJ plaque-like structures could be observed at the cell-cell interfaces. Cells expressing Cx50 or the mutant were also faithfully expressed GFP via an internal ribosome entry site (IRES) on this bicistronic expression vector (middle panels). The localization of the anti-Cx50 antibody labeling was also superimposed onto differential interference contrast image to show the cell morphology and location of the labelings. Nuclei were stained with bisBenimide H33258 (blue in all images). Scale bar = 20 μm.

None of the 23 cell pairs expressing D51M showed any GJ coupling in five independent transfections (Fig 4A). To explore the mechanism leading to this functional impairment, we fluorescently labelled D51M and Cx50 with anti-Cx50 C-terminal peptide antibody in transiently transfected HeLa cells. The antibody labels of Cx50-expressing cells were highly concentrated around the nuclei and were also shown as punctate localizations in intracellular compartments and at the cell-cell interfaces forming GJ plaque-like structures (Fig 5). This localization pattern is similar to those of Cx50 localizations reported earlier [37,38]. However, D51M showed a diffused intracellular localization without any visible localization at the cell-cell interfaces (Fig 5), suggesting that the functional impairment of this mutant was likely due to an impairment in cellular distribution. On average, the level of expression of D51M was apparently lower than that of Cx50 in cells expressing these vectors. Anti-Cx50 antibody positive cells were correlated well with GFP positive cells (Fig 5), indicating that our Cx50-IRES-GFP and D51M-IRES-GFP vectors faithfully express both Cx50/mutant and GFP in the transfected cells (Fig 5).

### Single channel properties of the Cx50 E1 mutants

**Fig 6:**
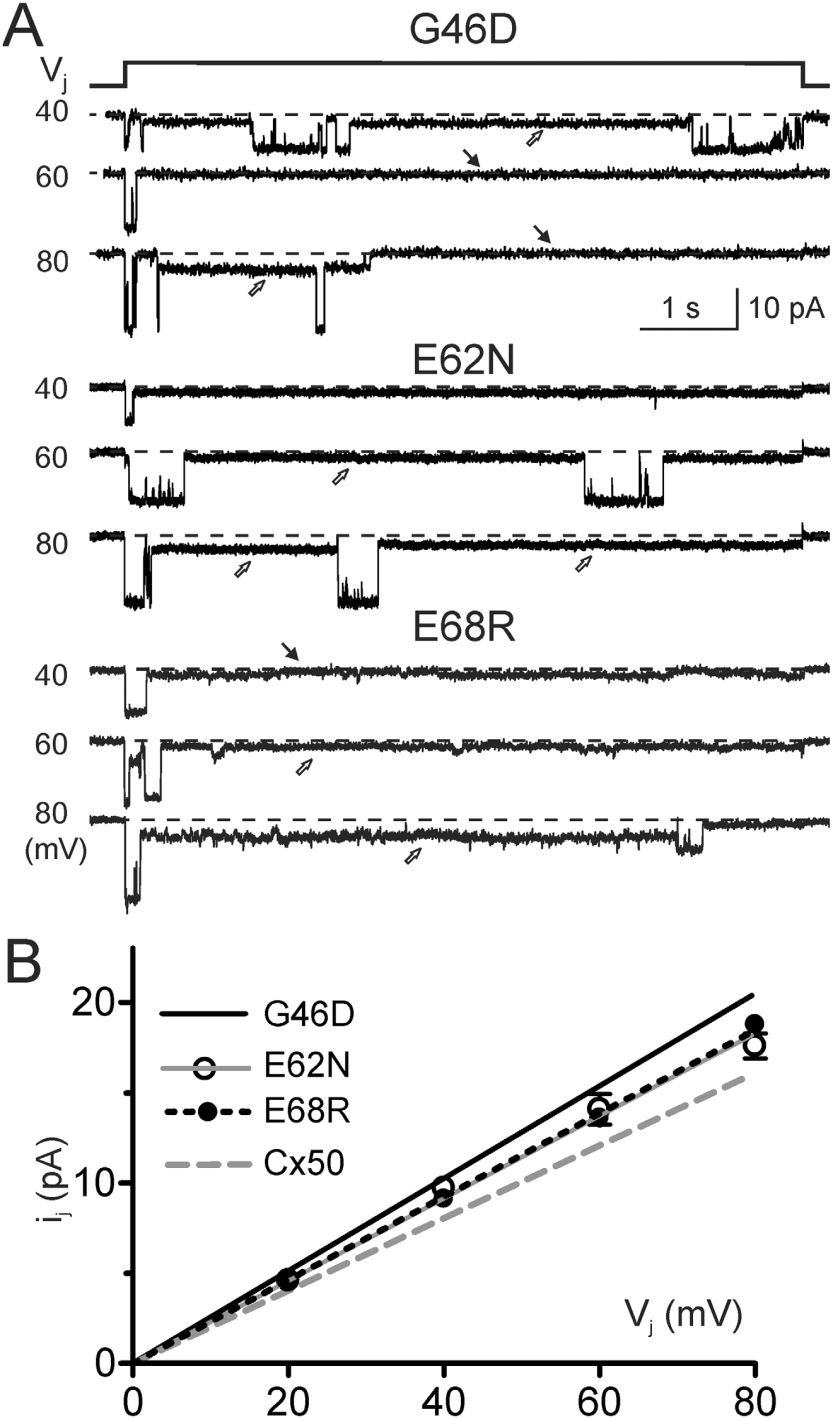
Unitary channel conductance of G46D, E62N and E68R channels were higher than that of Cx50. **A)** The i_j_ of G46D, E62N and E68R channels are illustrated in response to the V_j_s indicated. Main conductance state, one or more subconductance states (open arrows) and closed state (black arrows) are observed in these homotypic mutant channels. **B)** Linear regressions of i_j_−V_j_ plots of E62N (grey line) and E68R (black dashed line) were used to obtain the slope unitary conductance (γ_j_) of these mutant channels. The γ_j_s of these mutants are significantly larger than that of Cx50 (grey long dashed line), but lower than G46D. The regression lines of Cx50 and G46D are from [29].

**Fig 7:**
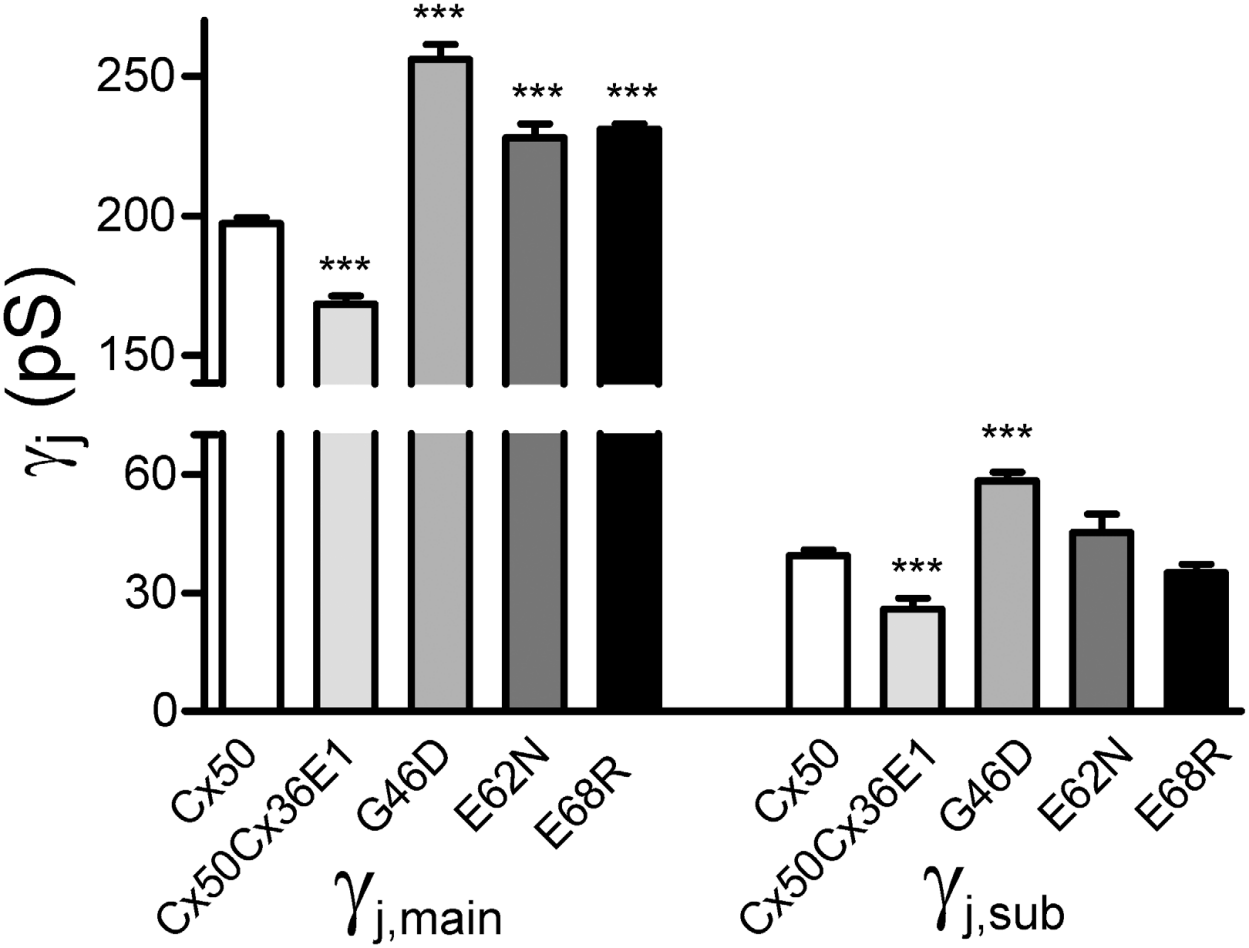
The main and subconductance levels of unitary channel conductance of gap junctions formed by Cx50Cx36E1, G46D, E62N or E68R. The main single channel conductance state (γ_j,main_) and the dominant subconductance (γ_j,sub_) of each mutant are shown. Statistical differences to those of wildtype Cx50 GJ channel are shown. The γ_j,main_ of Cx50 and G46D are obtained from [29].

Representative unitary channel currents (i_j_s) of G46D, E62N and E68R in response to V_j_ pulses (40, 60 and 80 mV) are illustrated in Fig 6A. All of these mutant channels displayed main open state, one or more subconductance states (open arrows) and in some mutants (G46D and E68R) fully closed state (black arrows) during the V_j_ pulses (Fig 6A). We used linear regressions of i_j_−V_j_ plots to obtain the main open state slope γ_j_s of G46D (Fig 6B, 256 ± 5 pS, data from [29]), E62N (228 ± 5 pS, n = 14, open circles) and E68R (231 ± 2 pS, n = 4). All of these γ_j_s are significantly larger (P < 0.001 in all cases) than that of wildtype Cx50 channel measured under the same conditions (201 ± 2 pS) [29]. In addition to the main open state, the channels of each mutant were also found to reside in at least one subconductance state. The conductance of the dominant subconductance state (γ_j,sub_) at ± 80 mV V_j_ of E62N (45 ± 5 pS, n = 6) and E68R (35 ± 2 pS, n = 4) were not different from the γ_j,sub_ of Cx50 (39 ± 1 pS, n = 6), while the γ_j,sub_ of G46D (58 ± 2 pS, n = 5) was significantly higher than that of Cx50 (P < 0.001). A bar graph was constructed to show the average of main conductance and subconductance of the chimera and mutants and the statistical significance comparing to those of wildtype Cx50 GJ channel (Fig 7).

### Structural and electrostatic properties of the Cx50Cx36E1 chimera and E1 point mutant channels

Homology models were used to relate the functional differences observed in the chimera and E1 point mutants to changes in channel size and electrostatics. Sequence identity between mouse Cx50 and human Cx26 is 57% in the domains resolved in the Cx26 crystal structure (PDB ID 2ZW3) [20]. The E1 domains of Cx50 and Cx36 show an even higher sequence identity (79% and 67%, respectively) with the Cx26 E1. These high levels of identity and complete lack of any deletions between the template and model sequences facilitated the creation of reliable homology models using the Cx26 crystal structure as a template. The Cx50Cx36E1 and the E1 mutant models were constructed the same way as described earlier [29] and retain the same backbone structure of the Cx26 template.

**Fig 8:**
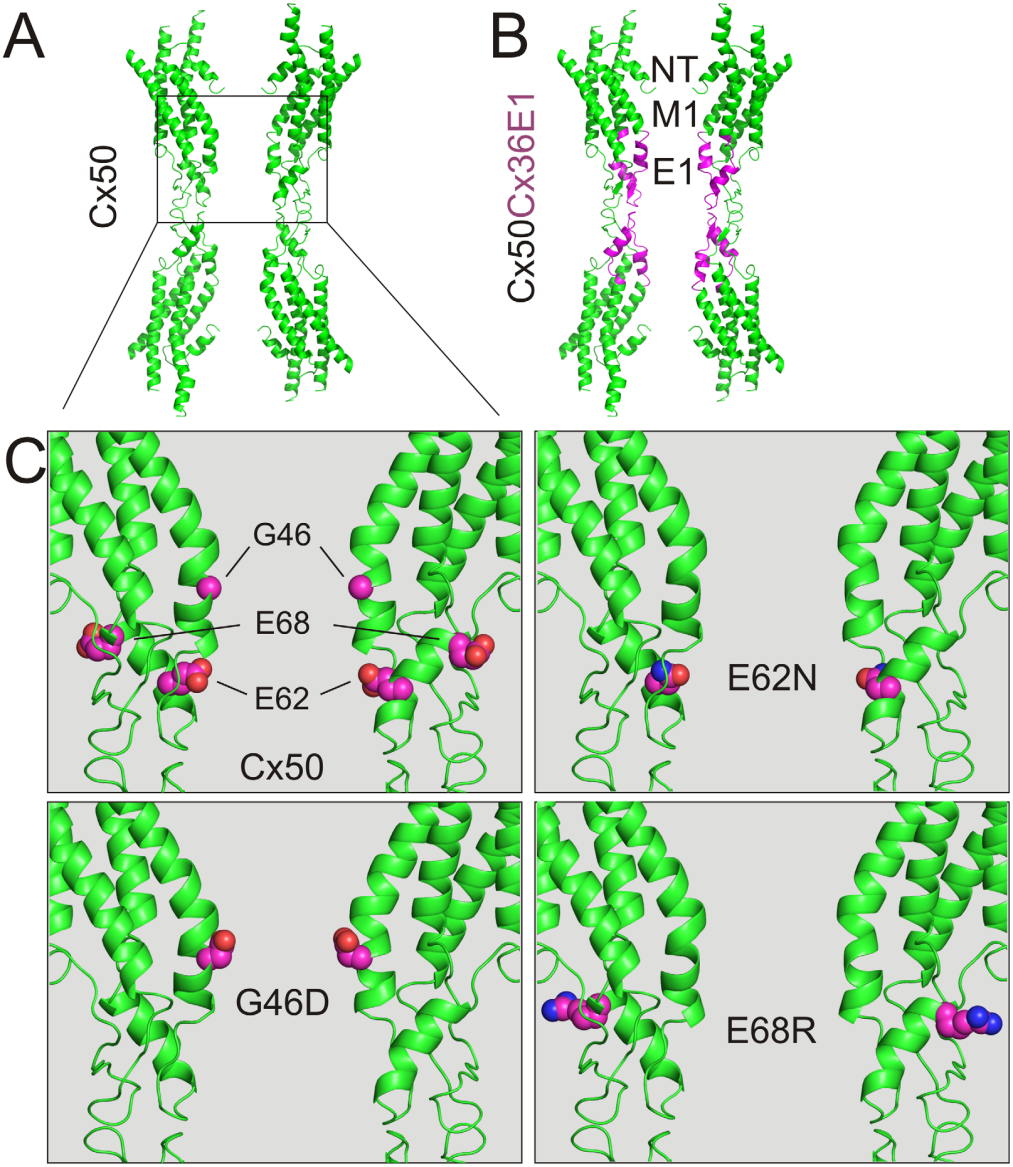
Homology models of Cx50Cx36E1 and Cx50 E1 mutants. **A)** A side view of the homology structural models (cartoon view and only 4/12 subunits are displayed) of Cx50 channel. **B)** A side view of Cx50Cx36E1 homology model (the E1 domains were displayed in magenta). Pore lining domains include amino terminals (NT), the first transmembrane (M1) and the first extracellular (E1) domains. **C)** An enlarged portion of Cx50 and the E1 mutant channels are shown. The side chains of the mutant residues are illustrated as spheres before (Cx50, top left panel) and after the mutation (G46D, E62N and E68R) as indicated.

The Cx50 and Cx50Cx36E1 models are shown in Fig 8A and 8B, respectively (only 4 subunits on each side are shown). The switched E1 domain (from Cx36) was colored in magenta to show that E1 contributes pore lining together with amino terminal (NT) and the first transmembrane (M1) domains (Fig 8B). An enlarged view of Cx50 E1 was used to show the locations of three residues to be mutated (Fig 8C). Among them, both G46 and E62 are pore-lining residues with their side chains toward the pore lumen, while the E68 and its side chain are not located at the pore surface. In addition, G46 is located at one of the narrowest part of the pore and E62 is situated at a position with a wider pore diameter. Depending on their locations in the pore, mutations of these residues can change both the local pore diameter if the side chain size is changed and the electrostatic properties if the side chain charge and polarization properties are changed.

**Fig 9:**
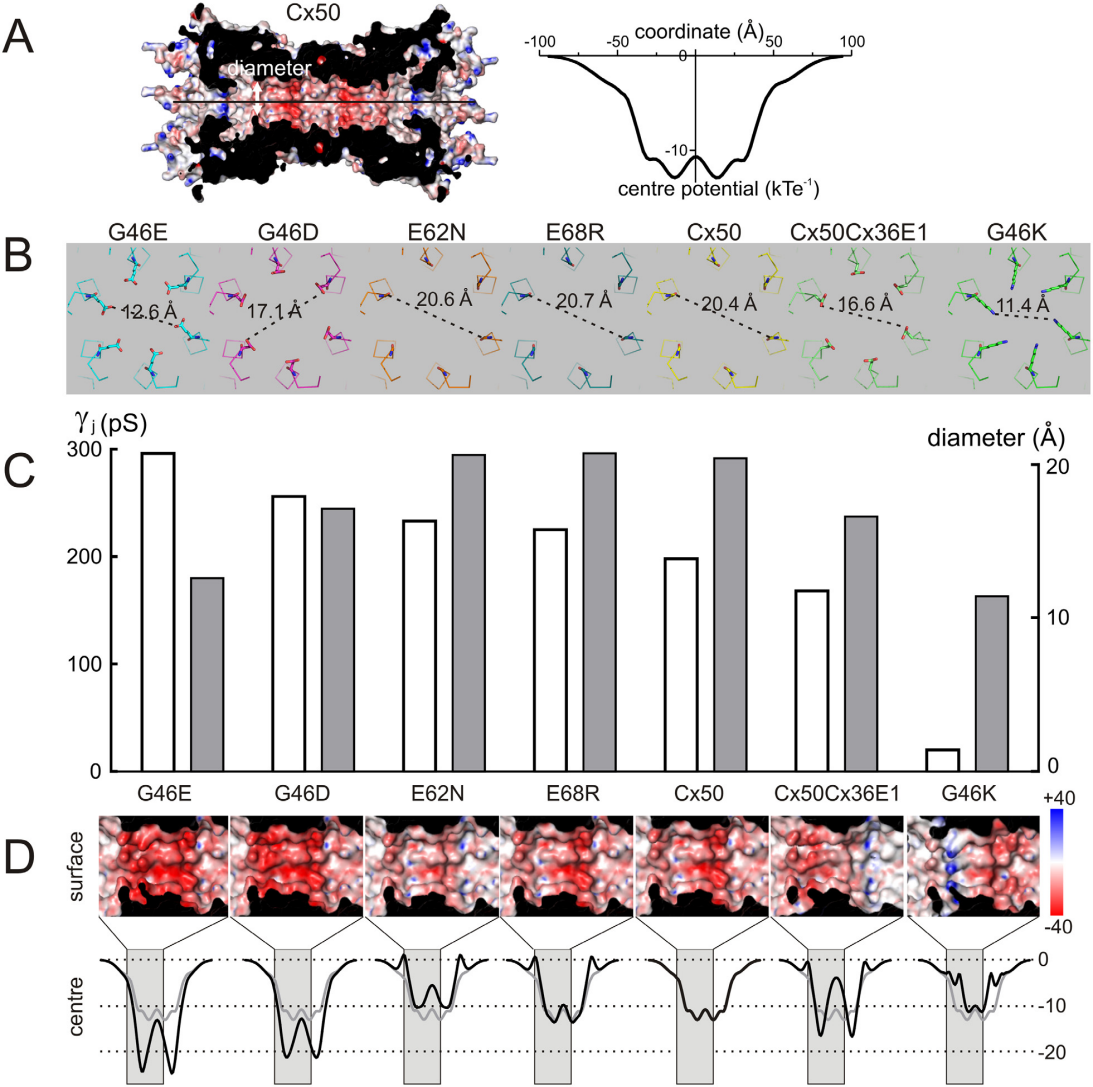
Homology models of the Cx50 E1 mutants. **A)** A side view of a cut open Cx50 channel is illustrated to show the pore surface electrostatic potentials (calculated with APBS) using dielectric constants of 2 (protein) and 80 (solutions) [33]. The pore centre electrostatic potential level of Cx50 GJ channel against the length of the pore was plotted (right panel). Negative potentials peaked at the E1 domains were observed for this Cx50 channel. **B)** A top view of homology models of Cx50 E1 mutants to show the predicted diameters at the 46^th^ residues (the narrowest part of the pore at E1). **C)** The single channel conductance (γ_j_) values of Cx50 E1 mutant channels (open bars) were plotted with the pore diameter at the 46^th^ residue (grey bars). **D)** Electrostatic potentials calculated from each of these mutant models are shown at the pore surface (top) and centre of the pore (bottom). Note that only part of the surface electrostatic potential (contain E1 domain) is shown as indicated (light grey areas in the centre electrostatic potential plots). The Cx50 channel electrostatic potential in the centre of the pore is superimposed on all of the mutants for direction comparison (grey lines).

To evaluate the changes in predicted pore diameter on the γ_j_, we measured the narrowest part of E1 from the channel models of chimera and the E1 mutants. For the purpose of comparison, we also included two additional E1 mutants (G46E and G46K) which have quite different effects on channel properties [29]. We plotted the γ_j_s with the predicted pore diameter at the 46^th^ residue, the narrowest part of the pore E1 domains (Fig 9B and 9C). No apparent correlation was observed between these two parameters. A simple explanation is that the pore diameter at the 46^th^ residue in these mutants is relatively large compared to the constriction at the NT domain [20].

The pore diameter in the region of the E1 domain could not explain the functional differences between the channels, and so we looked for differences in the electrostatic potential between the wildtype and mutant channels. The electrostatic potentials are presented in two fashions: in the first case, the molecular surface of the channel is colored according to the electrostatic potential at the solvent accessible surface, with a potential of +40 kTe^-1^ in blue transitioning to a potential of -40 kTe^-1^ in red (Fig 9A and 9D). From these surface potentials, it is clear that the overall potential in the region of the E1 domain is negative (Fig 9A). The negative electrostatic potential of the channel is also evident when potentials at the centre of the pore are plotted (Fig 9A, right panel). In the case of the wildtype Cx50 channel, the electrostatic potential in the centre of the pore reaches a minimum of approximately -12 kTe^-1^ close to the middle of the channel, where the extracellular domains are docked together. This region of negative electrostatic potential is affected by mutations in the E1 domain (Fig 9D). The G46D mutant showed a stronger negative surface potential than the wildtype channel, and the potential in the pore centre reached a minimum of -20 kTe^-1^. For a previously characterized G46E mutant, the surface potential was similar to the G46D mutant, but the potential in the pore centre was even deeper, with a minimum of -24 kTe^-1^. Both of these mutants displayed higher unitary channel conductance compared to the wildtype channel. In contrast, the Cx50Cx36E1 chimera had regions with a more positive surface potential compared to wildtype. In the pore centre, the minimum potential was somewhat lower than wildtype (-16 kTe^-1^), but there was a peak of positive potential in the middle of the channel. To take this analysis further, a previously characterized E1 mutant, Cx50 G46K was analyzed. In this case the surface potential was also more positive than the wildtype channel, and the region of negative potential in the centre of the pore was not nearly as extensive or as deep as wildtype. Thus, the electrostatic differences appear to correlate rather well with the changes in unitary channel conductance produced by the various mutations, with a large negative potential in the middle of the channel being associated with high values of conductance. The negative potentials in wildtype Cx50 channel might be important for the preferential permeation of cations over anions [23,29]. Along these lines, we predict that “constricted” regions of negative potential in the ion permeation passage near E1 domain, particularly in the case of the G46K mutant, will form an electrostatic barrier for cations moving through this cation-preferred channel.

## Discussion

The present study describes the effects of switching the entire E1 domain of Cx36 with that of Cx50 or switching only individual charge changed residues in the E1 on the V_j_-gating and single channel properties. Our results showed that the Cx50Cx36E1, G46D, E62N and E68R formed functional GJ channels with moderate, but not drastically changed V_j_-gating properties comparing to Cx50 GJ. However, the GJ channels formed by the chimera and these individual mutants displayed significant changes in unitary channel conductance (γ_j,main_). In addition, Cx50Cx36E1 and G46D GJ channels exhibit small, but statistically significant, differences in subconductance (γ_j,sub_) levels. Our homology models indicate that electrostatic potential field in the pore is an important parameter for the rate of ion permeation (γ_j,main_) in these E1 mutants. Our results are consistent with a proposal that E1 lines part of the pore and plays an important role in controlling the rate of ion permeation through this channel.

### Rate limiting factors for γ_j_ in Cx50 and the E1 mutant gap junction channels

Based on the previous studies in GJs of other connexins, we thought the differences in the E1 domains of Cx36 and Cx50 could play an important role in the quite distinct γ_j_ of these two GJs [24,25,26,27]. However, switching the entire E1 domain of Cx36 into Cx50 resulted in formation of functional chimera GJ channels with nearly identical V_j_-gating as Cx50 and only slightly reduced the γ_j_ from 201 to 168 pS (less than 17%). Interestingly, the point mutants in the Cx50 E1 (G46D, E62N and E68R) all showed an increased γ_j_, indicating that the E1 domain of Cx50 is not fully optimized for the rate of ion permeation. Based on our homology models of these and previously studied E1 mutants (G46E and G46K), we measured the diameter of the narrowest part of the E1 domain in these channel models and found that they are not correlated with the channel γ_j_, suggesting that the variations of the pore size caused by these mutants cannot account for the changes of γ_j_. To explore other factors important for ion permeation, we plotted the electrostatic potentials in the centre along the whole length of the channel and found that Cx50 and all mutant channels showed negative potentials at the E1 domains with large variations in the peak depth and width. High negative electrostatic potential favors accumulation of local cations [39,40], which represent the major ions moving through Cx50 channel [23,29]. Indeed mutant channels with the deepest and widest negative potential in the pore centre (G46E and G46D) showed the highest γ_j_s while the mutant with a relatively shallow and narrow negative potential (G46K) displayed the lowest γ_j_. Other mutant channels showed negative potentials similar to Cx50 and their respective γ_j_s were also largely similar to that of Cx50 channel. In summary, the negative electrostatic potential in the middle of the GJ seems to correlate qualitatively with the changes in γ_j_s caused by the mutations.

Comparing the pore centre and the pore surface electrostatic potentials, we found large disparities in G46K and Cx50Cx36E1 channels, both their surface electrostatic potentials showed high positive potentials (in blue color) in a discrete region in the E1 which could electrostatically repel cations away from the pore surface which could effectively reduce the local cation concentration. This could be an important additional factor for their lower γ_j_s especially for G46K.

In addition to the changes of the electrostatic potentials on the surface and in the centre of the pore, other factors might also contribute to the apparent lower γ_j_ in Cx50Cx36E1 than G46D, E62N and E68R GJ channels. 1) Several hydrophobic residues are different between Cx50 and Cx36, including a few at/near the inner pore position, e.g. F43T, W45Y, and S50T. The differences in their side chain size and orientation relative to the pore could alter the pore properties to reduce the rate of ion permeation. 2) There are ten simultaneously mutated residues in the chimera, if any single or combinations of these mutations could change the pore structure (main chain) to alter the pore size or property, then the resultant GJ channel could also have a changed property, such as a reduced γ_j_. An interesting observation from our study is that the chimera (with D51M together with other 9 mutations) was able to form functional GJs while the single mutant D51M was not. It is not clear on the relative contributions of these factors to the observed lower γ_j_ in the chimera than those individual mutant channels.

Among the Cx50 E1 mutants studied, the E68R mutation leads to a +2 increase in positive charge in the E1 domain, a larger change than the other mutants which involved changes of +1 or -1, but the E68R mutation had almost no effect on the functional properties of the channel. This can be explained by the location of the side chain of residue 68, which is present on the outside of the channel and therefore does not contribute to the electrostatics in the ion permeation passage. In fact, the lack of an effect of the E68R mutation on channel properties provides some assurance that our homology model is an accurate representation of the Cx50 channel structure. This is a simple explanation and needs to be further tested experimentally to be confirmed.

### Cx50 E1 chimera and mutant gap junction channels support contingent gating model

Transjunctional voltage dependent gating (V_j_-gating) is a universal property of GJ channels. Previous studies indicate that only pore-lining residues are able to sense the V_j_ changes [3,41], including the extracellular domain E1. V_j_ can gate single GJ channel to a substate or a fully closed state. The characteristics of these two gates are different, gating transitions from open to residue state are relatively fast with rise/decay time within a few milliseconds thus known as the fast gate (also known as V_j_-gate). While the gating transitions to fully closed state via either open or residue state are slower with rise/decay time longer than 10 milliseconds, thus this gate is called the slow gate. It is also known as ‘loop gate’ as the proposed domain responsible for this gate are extracellular loop domains. In wildtype Cx50 GJ channels the dominant V_j_-gating events are the fast gate especially at the V_j_s of ±60–100 mV. Slow gate rarely occurs, once observed it is usually very brief [23,42]. Different from wildtype Cx50 GJs, Cx50Cx36E1, G46D, and E68R GJ channels showed long lived closed state in the single channel current records. Perhaps that in the GJ channel of Cx50Cx36E1 and these Cx50 E1 mutants, the slow gate sensitivity and/or the stability of the closed state was increased. An alternative explanation would be that in these Cx50 E1 mutants the stability of residue/open state was changed. These possibilities are difficult to further discriminate as they have little impact on the macroscopic V_j_-gating parameters, such as G_min_ and slope *A* (which also reflect the aggregated gating charge). From our G_j,ss_ / V_j_ plots, we did not observe any consistent changes in the V_j_-gating parameters on both V_j_ polarities. The most drastic V_j_-gating property change was observed in Cx50 G46K channel, in which the G_min_ is much higher than that of Cx50 and the slope *A* (inversely correlated with the aggregated gating charge z) was significantly reduced. These changes are consistent with a model, that in G46K GJ channel only the slow gate works during V_j_ changes, the fast gate is either impaired or intact but is not operating due to insufficient V_j_ drop on the fast gate ‘sensor’. A substantial drop in the γ_j_s of G46K homotypic channel and heterotypic G46K/Cx50 channel indicate the V_j_ distribution in the pore are likely redistributed with more voltage drop across E1 G46K area, likely the ‘sensor’ location for the slow gate [29]. Our data support the contingent gating model of GJ channels and the gating properties of G46K channel and heterotypic G46K/Cx50 channel suggest that the slow gate in these channels are dominant and contingent elimination of the fast gate of both hemichannels [29].

### Residues in E1 domain of Cx50 and other connexins are hotspots for disease-linked mutants and play several important roles in gap junction function

Cx50D51 is a well conserved residue in several human connexins, including Cx26, Cx30, Cx30.3, Cx37, Cx40 and Cx46. It’s been found in oocyte expression system that Cx50D51 and Cx46D51 along with G46 in both connexins in the E1 domain line the channel pore, play a role in unitary hemichannel conductance and also in Cx50 hemichannel gating [14,16]. More interestingly, skin disease-linked mutations were found on the equivalent residue in Cx26, D50N and D50Y, impairing calcium regulated hemichannel gating (leading to excessive opening of hemichannels) possibly due to a change in inter-subunit interactions [17,18,43]. Whether in Cx50 the D51 also play a role in inter-subunit interactions is unknown. If it did, one of the proposed HB formation between D51 and Q49 of neighbor subunit would be possible, but at the Cx26K61 equivalent position in Cx50 is Asp (D62), which would not be able to form salt bridge with D51 as both of them carry negative charges. We observed that Cx50 D51M was unable to form any functional GJ channels and the localization study with antibody labeling indicate that D51M was diffusely distributed in the cytosol and never form large clusters at the cell-cell interfaces in our mammalian model cells (both HeLa and N2A cells). We do not know if this mutant can assemble into connexons and traffic to the plasma membrane, but if it could, they must be at an extremely low level that beyond the detection threshold of the antibody used. It is not surprising that no GJ function was observed in cell pairs expressing Cx50D51M. Several other mutations in the equivalent residue in Cx26, such as D50N, D50A and D50C, also failed to make GJ channels, even that hemichannels are readily identifiable on the cell plasma membrane in *Xenopus* oocyte [18,44]. It is interesting to note that Cx26 D50A also showed an apparent localization defects in HeLa cells and in keratinocytes [44], indicating a similar role of this residue in both Cx50 and Cx26.

Missense mutations on the 46^th^ position of Cx50, G46V and G46R, were found to link to cataracts [38,45]. In vitro expression of G46V showed a substantially elevated hemichannel activity, which could play a role in cell death leading to disease [37,38]. Several studies on a keratitis-ichthyosis-deafness syndrome-linked mutant in Cx26, G45E (a position equivalent to G46 of Cx50) showed an increase in hemichannel unitary conductance, an altered V_j_-gating and an increase in hemichannel function [46,47,48]. The hemichannel conductance increase in Cx26 G45E is consistent with our data on an increased γ_j_ of Cx50 G46E (or G46D) [29]. However, we did not see a substantial change in V_j_-gating properties in these two mutant GJs.

In addition to the D51 and G46 positions in Cx50, several other mutants were also reported on the E1 domain of Cx50, such as V44E, W45S, D47N/Y, E48K, V64G, S73F and V79L [49]. Functional studies on V44E, D47N and V79L showed that each of these mutants displayed distinct impairments in the γ_j_, V_j_-gating or macroscopic coupling conductance [15]. W45S showed impairment on both GJ and hemichannel function [37]. These disease-linked mutant studies together with structure function studies on the key residues in the E1 domain of Cx50 [16,50] indicate that the E1 is a critical domain serving several important functions in GJ or hemichannel regulation.

In summary our experimental data on Cx50 E1 chimera/mutants and our homology models indicate that switching the entire E1 domain or the individual charge changed residues (except D51M) from Cx36 into Cx50 resulted in minor to moderate changes in V_j_-gating properties. It was not surprising to observe a significant decrease in γ_j_ in the Cx50Cx36E1 chimera, but a substantial increase in γ_j_ in G46D, E62N and E68R was unexpected. Our homology models indicate that the Cx50 E1 domain lines the pore and can facilitate/limit the rate of ion permeation by a combination of pore size and their electrostatic properties.

## Supporting Information

S1 File Electrostatic Analysis of Gap Junction Channels. The homology model of the Cx50 gap junction channel is supplied as a PDB file. This file can be used to generate a three dimensional map of the electrostatic potential using the “Advanced Poisson-Boltzmann Solver” (APBS) [33] that is integrated into the molecular graphics program "PyMol”. Output from this program is the “pymol-generated.dx” file that contains electrostatic potentials at defined grid points. The supplemental file “potential.net” script can be processed by OpenDX and will produce a graphical output along with a table (z-data) containing electrostatic potentials along the z-axis. To run the “potential.net” script, replace three instances of "PATH" with your working directory that contains the appropriate pymol-generated.dx file. Data available from the Dryad Digital Repository: http://dx.doi.org/10.5061/dryad.98d35.
(NET)

## Abbreviations

Cx50: connexin50
E1: the first extracellular domain
G_j,ss_: normalized steady-state junctional conductance
γ_j_: unitary channel conductance
I_j_: macroscopic junctional current
i_j_: single channel junctional current
V_j_: transjunctional voltage
